# A Retrospective Study Using a Novel Body-Shift Implant Design with a Novel Alloplastic Particulate Grafting Material in Immediate Extraction Sockets

**DOI:** 10.1055/s-0045-1801849

**Published:** 2025-02-03

**Authors:** Dominic B. O'Hooley, Costa Nicolopoulos, Mark G. Worthing, Petros Yuvanoglu, Fotis Melas, Peter J. M. Fairbairn, Gregori M. Kurtzman

**Affiliations:** 1 Private Practice, London, United Kingdom; 2 Private Practice, Athens, Greece; 3 Department of Periodontology and Implant Dentistry, University of Detroit Mercy School of Dentistry, Detroit, Michigan, United States; 4 Department of Oral Surgery, Tufts University School of Dental Medicine, London, United Kingdom; 5 Private Practice, Silver Spring, Maryland, United States

**Keywords:** novel implant design, alloplastic graft materials, immediate tooth replacement

## Abstract

With resurgence in immediate tooth replacement therapy (ITRT) as a method of preserving both hard and soft tissues for improved aesthetic outcomes, this multicenter, prospective study looked at two novel products and their effect on those outcomes. Thirty-one maxillary single-tooth implants were included, of these 54.8% were central incisors, 25.8% lateral incisors, and 19.4% canines. Three complications were reported; one case nondraining fistula, one case a nonseated provisional restoration, and one case a fractured zirconia abutment. The definitive restorations were delivered between 4 hours and 18 months postimplant placement and all restorations were screw-retained. ITRT is frequently utilized when a tooth to be extracted will be replaced by an implant aiding in preservation of the hard and soft tissue that may be lost due to resorption during healing of the extraction socket. The narrower neck region of the Inverta implant results in thicker crestal bone around the implant, where loading under function occurs. Grafting that area around the implant at placement with EthOss results in more predictable bone stability in the long term.

## Introduction

Immediate tooth replacement therapy (ITRT) is a method of preserving both hard and soft tissues for improved aesthetic outcomes when an implant is planned for a failing tooth. 1 This is especially important in the anterior region in both maxilla and mandible. Specifically, in the maxillary anterior region, tooth extraction frequently leads to significant hard tissue changes, which in turn affect the soft tissue. ITRT following extraction reduces bone loss and the subsequent soft tissue changes that affect aesthetics also shortening overall treatment time. 2 The crestal bone thickness has been noted to be thin specifically on the labial aspect and decreases as the patient ages related to periodontal changes. 3 Yet, ITRT following extraction poses some clinical challenges related to the thickness of the crestal bone around the implants neck. 4 Standard design implants have a neck region that typically parallels the implant's body to achieve initial implant stability. This results in thin crestal bone at placement, which may lead to resorption during healing and osseointegration or underfunction over time. A recently published study recommended when ITRT is planned at the anterior that narrower diameter implants be utilized to allow thicker bone following healing of the jump gap. 5 Yet, this has the potential to limit initial implant stability as the crestal portion of the implant may not be in contact with the extraction socket walls, limiting the ability for immediate provisionalization of the implant. It is accepted that a jump gap of 2.0 mm or greater requires osseous grafting at implant placement to avoid resorption of the crestal bone during the healing phase. 6 Utilization of an implant that has contact with the crestal bone specifically the labial aspect of the ridge crest and has a jump gap of less than 2.0 mm does not require grafting at implant placement. But the thin labial bone may resorb during site healing or later underfunction. A novel body-shift implant design has been introduced to enable high primary stability in immediate extraction sockets to create thicker crestal bone following site healing and can aid in preserving that bone in the position related to the implant's length. This, when combined with a novel alloplastic bone augmentation material placed into the circumferential jumping gaps of said extraction sockets, yields thicker bone following site healing.

The prospective study presented reviewed single-tooth ITRT in the maxillary anterior (incisor and canine) region in both intact and labially deficient extraction sockets. Those immediate implant placement sites were treated with circumferential jumping gap augmentation with the novel alloplastic material at implant placement into the extraction socket. This multicenter prospective study presents 1 year plus data from a prospective single-arm cohort study.

## Methods

The clinical and radiographic outcomes of 31 ITRT implants were evaluated; using a novel body-shift implant design to preferentially engage the residual bony volume of the socket walls after immediate tooth extraction aims to optimize apical primary stability for ITRT. 7 8 Patients were included where it was determined that an immediate implant could be placed at the time of extraction. Those patients where immediate placement could not be performed due to the clinical situation were excluded from the study group.

A multicenter registry was created for this retrospective multicenter study using the novel body-shift implant design in conjunction with the novel alloplastic bone regeneration material. Registered patients provided consent in accordance with the Declaration of Helsinki. 9 Four study centers, three in the United Kingdom and the other in Dubai, the United Arab Emirates, participated in the data collection and approval. The data was extracted from the multicenter registry based upon the following criteria:

1. Single-tooth ITRT in the maxillary incisor and canine regions.
2. Treatment with the novel alloplastic bone augmentation material in types I, II, IV-A, and IV-B sockets. 10
3. Cone-beam computed tomography (CBCT) evaluation using standard dental CBCT scanning units. 11 12

**Fig. 1:**
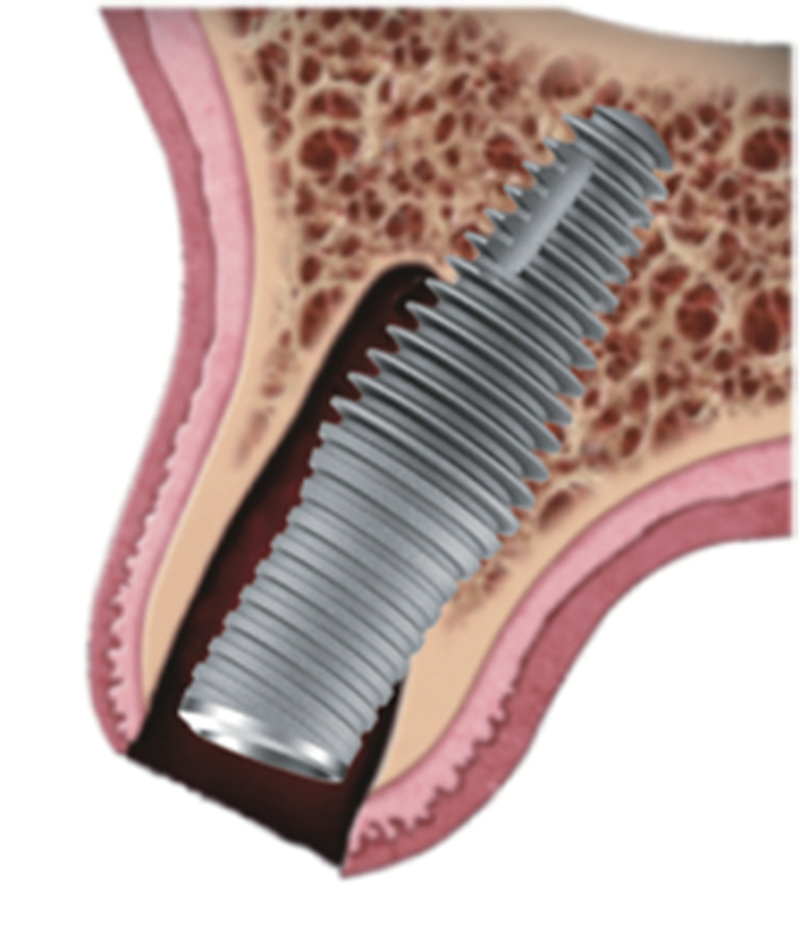
Illustration of the Inverta implant orientation associated with an extraction socket.

Within the study group, following extraction, if fenestration/dehiscence was noted, angulation of the implant was altered to eliminate the defect being present following implant placement. Utilization of the Inverta implant with its 12-degree angle correction aided in facilitating the procedure. The implant was introduced in 2018 (Inverta, Southern Implants, South Africa) combining an internal prosthetic angle correction of 12 degrees and a body-shift feature ( Fig. 1 ). These variations in diameter, three-dimensional (3D) profile, and thread pattern within a single implant design focused on optimizing primary apical stability. Additionally, the relatively narrow coronal portion of this implant design increases the labial jumping gap compared with traditional tapered implants with a comparative apical volumetric profile. Combining this with an internal angle correction of 12 degrees (Co-Axis), allows implant placement within the maximal residual bony ridge volume, with both the aim of optimizing bone to implant contact, implant head position, and creation of a circumferential jumping gap. 13 14 This maximizes coronal distance between the implant's coronal portion and adjacent osseous structures. Thus, allowing ideal 3D implant head positioning for prosthetic emergence and larger volume for circumferential placement of grafting materials, both aiding in preservation and maintenance of ridge architecture. 7 15 16 17 The implants surface uses a moderately rough, sand-blasted microsurface topography 7 ( Supplementary Fig. S1 , available in the online version only).

A novel alloplastic bone augmentation material was introduced in 2015 (EthOss, EthOss Regeneration Ltd, United Kingdom), which is composed of 65% beta-tricalcium phosphate (β-TCP) (Ca3(PO4)2) and 35% calcium sulfate (CS) (CaSO4). This fully synthetic particulate material has been shown to result in over 50% host bone at 12 weeks with only 10 to 12% residual graft material. Full resorption is noted at 6 to 12 months in line with extensive published material on porous β-TCP but may vary due to patient physiology. 18 19 That resorption is synchronous with new bone formation and in line with other CaP materials, 20 21 and has an osteoinductive potential to upregulate the host regeneration. 22 β-TCP has been reported to fully resorb, 22 23 24 and host bone provides an ideal foundation for long-term health and stability of the hard/soft tissue complex. 25 26 This is particularly critical in the aesthetic zone.

No cohort study has yet investigated combining this novel implant design and alloplastic material, for ITRT. 10 Therefore, the aim of this study was to present 1 year plus data from a retrospective, single-arm, multicenter study that correlated clinical and radiographic outcomes.

### Clinical Procedure

The surgical treatment protocol involved minimally traumatic tooth extraction utilizing elevators and forceps following standard procedures. Residual socket debridement was performed using both degranulation burs and sharp curettage. The osteotomy was undersized by at least 0.5 mm in circumference to allow placement of the novel design of the body-shift implant of case-specific choice, 3.0 to 5.0 mm from the labial-free gingival isthmus margin. A minimum immediate primary stability of 35 Ncm was required to facilitate immediate full contoured provisional restorations in nonocclusion. 27 The circumferential jumping gap was filled with the novel alloplastic bone augmentation material (EthOss). A screw-retained provisional restoration was fabricated from direct pick up of a Polyetheretherketone (PEEK) or titanium temporary abutment (Southern Implants PTY). These were adjusted to nonocclusion in centric and excursions. The emergence profile of the provisional screw-retained restorations is not only important for particulate graft retention but also to adapt and develop soft tissue emergence contours. 28 The Cervico system (VP Innovato Holdings Ltd, Cyprus) was used by one study center, while free-hand or in-house laboratory-manufactured provisional shell crowns were used by the other centers. A concave subgingival emergence profile was obtained in all cases.

### Case Examples

#### Case 1

**Fig. 2:**
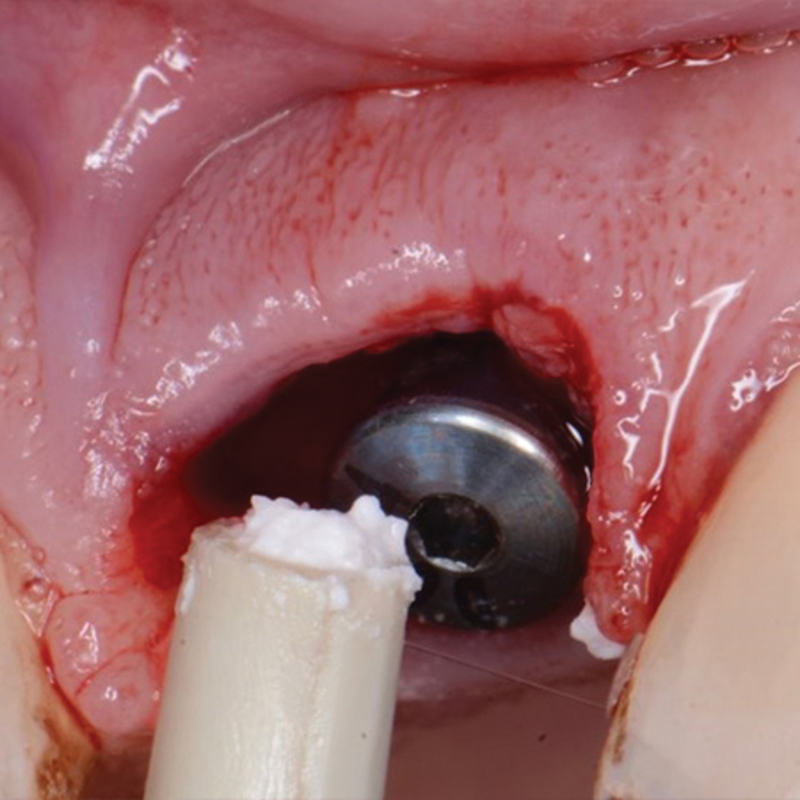
EthOss placement into the circumferential jump gap between the implant and extraction socket wall at implant placement.

**Fig. 3:**
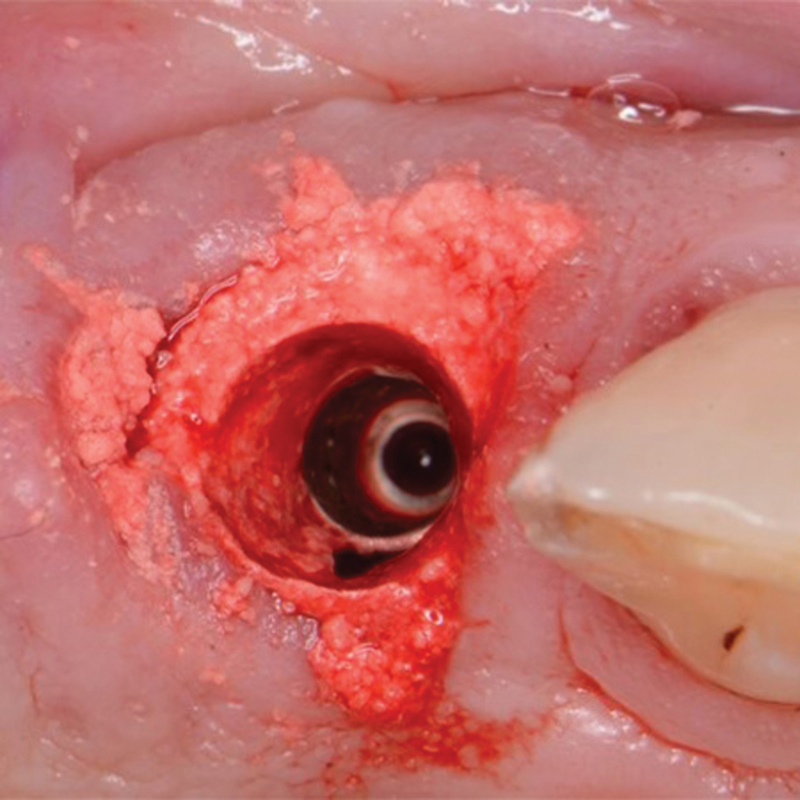
EthOss placed in the circumferential jump gap after provisional restoration was removed to allow cleanup of excess graft material during the surgery.

**Fig. 4:**
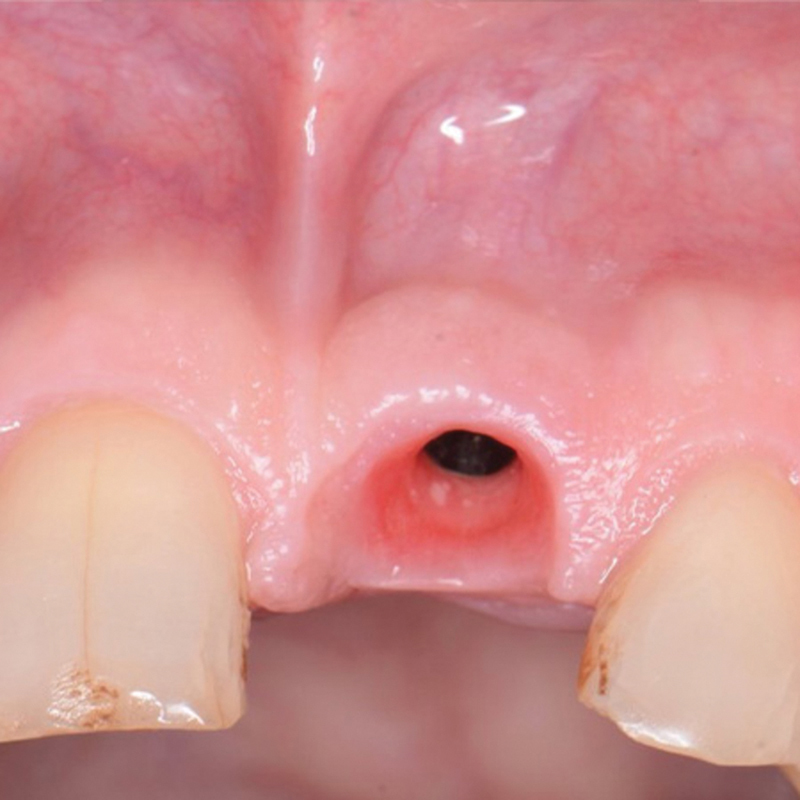
Gingival architecture following removal of the provisional restoration prior to delivery of final restoration demonstrating healthy soft tissue and a lack of inflammation.

**Fig. 5:**
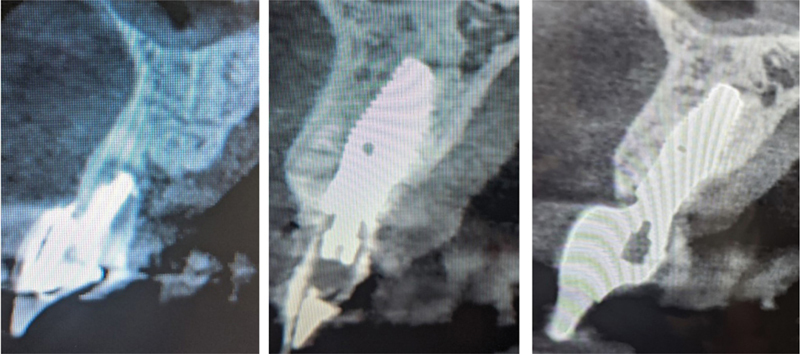
Cone-beam computed tomography (CBCT) cross-sections at the preoperative of the failing tooth (left), immediately following immediate implant placement, and provisional restoration placement (middle) and following 12 months' loading and final restoration placement (right).

A failing left maxillary central incisor that had previous endodontic treatment and had been restored with a post/core and crown presented to one of the centers ( Supplementary Fig. S2 , available in the online version only). The ceramic crown was fractured on the facial cervical. A CBCT was taken and in cross-section, it was noted that the tooth demonstrated fracture in the root and had minimal labial ridge present over the root (see Fig. 5 , left). Treatment recommendation was extraction and immediate placement of an implant with labial socket grafting in the jump gap and placement of an immediate provisional restoration. The patient accepted treatment and consent forms were signed. The tooth was atraumatically extracted under local anesthetic in a flapless approach. An Inverta Co-Axis implant was placed into the extraction socket ( Supplementary Fig. S3 , available in the online version only). Utilization of the Co-Axis version allowed the prosthetic screw axis to be placed on the lingual side of the center of the ridge ( Supplementary Fig. S4 , available in the online version only). A screw-retained provisional restoration was fabricated on a Polymethyl methacrylate (PMMA) abutment intraorally ( Supplementary Fig. S5 , available in the online version only). The provisional restoration was removed, and a healing abutment was placed and then the EthOss graft material was placed to fill the jump gap around the narrower Inverta coronal section of the implant ( Fig. 2 ). The healing abutment was removed, and excess graft material removed from the site ( Fig. 3 ). The screw-retained provisional restoration was placed and occlusion checked to remove any contact in centric and excursive movements ( Supplementary Fig. S6 , available in the online version only). A CBCT was taken to document the labial aspect of the implant (see Fig. 5 , middle) The patient returned after 4 months for initiation of the final restoration. The provisional restoration was removed, and the soft tissue demonstrated healthy soft tissue cuff with no inflammation noted ( Fig. 4 ). The final screw-retained restoration was fabricated and inserted at another appointment. Aesthetically, it replicated natural ridge architecture and blended with the adjacent ridge at the natural dentition ( Supplementary Fig. S7 , available in the online version only). A CBCT taken at 12 months of implant placement demonstrated stability of the grafted labial aspect of the ridge in relation to the implant ( Fig. 5 , right).

#### Case 2

A patient presented with pain on the maxillary right lateral incisor that had been previously treated with endodontics and restored with a post/core and crown. Clinically, the labial gingiva presented with a dark appearance and was inflamed ( Supplementary Fig. S8 , available in the online version only). A periapical radiograph was taken, and a horizontal fracture was noted supracrestally with moderate crestal bone loss on the root ( Supplementary Fig. S9 , available in the online version only). The patient was informed of the clinical findings and the recommendation of extraction with immediate implant placement and an immediate provisional restoration. The patient accepted the treatment recommendation, and the consent form was signed. As with the previous case example, the tooth was extracted atraumatically and an immediate Inverta implant was placed. The jump gap was filled with the EthOss graft material, and a screw-retained provisional restoration was placed out of occlusion. Following a 6-month healing period, the provisional restoration was converted to a final screw-retained restoration. During the 12-month period, the ridge was noninflamed, and contours matched the adjacent labial aspects of the ridge ( Supplementary Fig. S10 , available in the online version only). The gingival tissue was healthy and had natural aesthetics ( Supplementary Fig. S11 , available in the online version only).

### Data Collection

The following data points were evaluated for this study. Mean values and standard deviations were calculated for each category.

### Clinical Evaluation

#### Implant Primary Stability

At the time of implant placement, the insertion torque values were recorded in Newton centimeters (Ncm) using either an electric handpiece or manual surgical torque wrench.

#### Pink Esthetic Score

The pink esthetic score (PES) is a method for evaluating the appearance of soft tissue around implant-supported single crowns. The PES assesses seven variables, including the mesial and distal papilla, soft tissue level, contour, color, texture, and alveolar process deficiency. High-resolution images were captured using digital single-lens reflex cameras with 105-mm macrolens and ring flash system at 1:1 ratio. Images were rated by the four observers and all measurements were made twice, at least 24 hours apart. Both immediate preoperative and a minimum of 12-month (range 12–50 months) postoperative images were taken and evaluated. 29

### Radiological Evaluation

#### Labial Plate Dimension

The presence and width of the labial plate was measured prior to ITRT in a CBCT cross-section and at least 12 months (range 12–20 months) afterward. Measurements were taken (in mm) at one level: the implant–abutment interface equivalent to the midfacial labial plate bone crest. 30 31 At this level, two reference points were defined; (1) the outermost aspect of the labial bone plate and (2) the first radiographic bone-to-implant contact point connected by a straight line perpendicular to the implant body. The distance between the two points was measured using proprietary CBCT digital imaging software (Carestream Dental Imaging Software).

## Results

Thirty-one maxillary single-tooth implants were included, based on the previously described criteria. The mean patient age was 59.25 years (range 24–79 years) with 17 male and 8 female patients. Of these 31 included implants, 54.8% were central incisors, 25.8% lateral incisors, and 19.4% canines ( Supplementary Table S1 , available in the online version only). The reasons for tooth extraction included root fracture, caries, periodontitis, and unretrievable fractured posts. The circumferential labially aspect of the extraction socket following implant placement emphasized jump gaps that were all grafted with β-TCP/CS alloplast (EthOss) at the time of implant placement. Three complications were reported; one case nondraining fistula, one case a nonseated provisional restoration, and one case of a fractured zirconia abutment. These complications did not affect the final results and were included in the study group. The definitive restorations were delivered between 4 hours and 18 months postimplant placement. All restorations were screw-retained ( *n* = 31). The mean insertion torque value was 58 Ncm with a range of 10 to 100 Ncm.

### Radiographic Evaluation

Thirty-one sets of CBCTs were taken at the time of ITRT, and at least 12 months after loading were available for radiographic evaluation. The mean lateral bone thickness (LBT) at the time of ITRT was 0.7 mm with a range from 0 to 2.1 mm ( Supplementary Fig. S12 , available in the online version only). At follow-up of at least 12 months of implant loading, the mean LBT was 2.3 mm, with a range of 0.8 to 4.3 mm. This represents a mean increase in LBT of 1.7 mm ( Supplementary Fig. S13 , available in the online version only and Supplementary Table S2 , available in the online version only).

### Clinical Evaluation

The preoperative PES mean score was 10 with a range from 5 to 13. The postoperative PES mean at least 12 months after implant loading was 12 with a range from 10 to 14 ( Supplementary Table S3 and S4 , available in the online version only).

## Discussion

The optimal method to retain host tissues is through reduced surgery and hence the concept of ITRT was developed approximately 20 years ago with flapless immediate implant placement. 32 However, there were longer term issues with the initial iteration mainly due to the selection of larger tapered implants that closely adapted to the socket after extraction. 33 34 Discoloration (bluing) of the gingiva due to the gray body of the implant being visible through the reduced or entirely absent labial gingiva is an unfortunate negative sequela observed in thin tissue biotypes. This can be followed with possible gingival recession and frank metal exposure. These are but two common issues where the implant dimensions precluded retention of endosteal bone to support the labial cortical plate. 35 36

It is now the consensus 3 that we need to have an optimum implant coronal dimension to allow a buccal jump gap to graft to maintain and regenerate the buccal plate to ensure long-term soft tissue stability with attached keratinized tissue. 37 The added benefits of variable platform switching associated with subcrestal angled-correction implants has also been reported. 38

The use of a thin buccal root section in partial extraction therapy has also shown promising results in buccal plate preservation. This will be presented in a future study. 39

Recently, a novel implant design from Southern Implants, the Inverta, 3 has been released. Through its novel body-shift design, there is a narrow coronal portion to optimize the buccal jumping gap. This results in thicker crestal bone to better support the soft tissue and provide better long-term aesthetics. The apical tapered portion ensures high primary stability as required for immediate loading. The Inverta implant is offered with both a straight and a subcrestal angle correction version, termed Co-Axis, 40 which is a 12-degree angled internal connection to assist the treating surgeon with implant placement allowing optimized screw channel location. These unique aspects of the Inverta have multiple advantages for the surgeon and restorative dentist. There is an increased likelihood of correct 3D implant placement for high primary stability with screw retention as well as a buccally emphasized circumferential jump gap at the crestal region. This circumferential jump gap should be grafted at the time of implant placement for optimal results as reported in the literature. 41 42 Within this study, EthOss comprised of 65% β-TCP and 35% CS enables the material to “set” following placement, thus making it stable and proving a barrier function to soft tissue ingress during the initial healing period. 43 Hence, the requisite to use a separate collagen-type membrane can be dispensed with ensuring the optimal periosteal healing response. 22

There has been extensive research on guided bone regeneration 44 45 and the use of resorbable and nonresorbable membranes, 46 as well as on the ability of the host periosteum in bone regeneration. 47 48 49 For this reason, it is felt that the use of a membrane not only impedes host blood supply to the site with up to 50% fewer blood vessels in the new bone but may also impede the host periosteal induction of stromal cell-derived factors. 50 These bone morphogenetic proteins attract mesenchymal stem cells to the healing site, where they can differentiate into osteoblasts, thus regenerating new host bone. 51 52

The novel ability of this alloplastic bone regeneration material to not require an exogenous membrane is of greater importance in ITRT where membrane placement increases surgical difficulty. 53 54 The CS element in EthOss, additionally shows bacteriostatic properties 55 with an improved soft tissue healing response, which is beneficial in this protocol. A further benefit of the CS is it resorbs at 3 to 4 weeks depending on patient physiology and graft volume. This resorption creates new space between the β-TCP particles for neovascular ingrowth and a resultant upregulated angiogenesis. This upregulation of host bone regeneration has been recently shown in a new study using osteoprotegerinmarkers. 17 Immediate placement of the semiconductive titanium implant has in itself shown to upregulate host regeneration of bone and this in conjunction with earlier loading in function with the definitive restoration shows a further enhancement of upregulation via functional remodeling. 50

Studies have demonstrated that over 50% of new host bone results at 12 weeks postgrafting with around only 10% residual graft material at this time. 17 56 57 58 This results in new host bone earlier, 59 which is important for long-term stability of the hard and soft tissues. When the implant is in function, the bone will turn over and further improve, maintaining the profile in line with Wolff. 60

In this study, we are only measuring new buccal bone along with the PES of the soft tissue. The interproximal bone is also of great importance in regeneration as this will ensure the long-term stability of the papillae. 61 This will be investigated in further studies. Limitations of this study are the number of study participants and further studies would allow a larger number of cases to be compared and reinforce the results presented in this study.

## Conclusion

This study has exhibited the value of the unique characteristics of these two products to enhance the success and viability of the ITRT protocol. The relatively narrow coronal portion of the Inverta implant along with the regenerative potential of EthOss appears to have improved bone regeneration in the critical aesthetic coronal zone for enhanced tissue stability.

Although this multicenter study appears to show synergy between both this novel implant and graft material, further studies are required to investigate longer term stability of the treated sites, with concentration on the interdental bony septum and the effect of partial extraction therapies. Additionally, a future study comparing the novel implant presented in this study with traditional implant designs would add more evidence to the concepts presented in this article regarding socket grafting at implant placement in immediate sites in the critical aesthetic coronal zone.
