# Supplementary material for: A Retrospective Study Using a Novel Body-Shift Implant Design with a Novel Alloplastic Particulate Grafting Material in Immediate Extraction Sockets
Source: Eur J Dent. 2025 Feb 3;19(3):860–7. doi: 10.1055/s-0045-1801849 (PMC12182418; doi:10.1055/s-0045-1801849)
Supplement: Supplementary file 1 — Supplementary Material [file 10-1055-s-0045-1801849-s2493788.pdf]

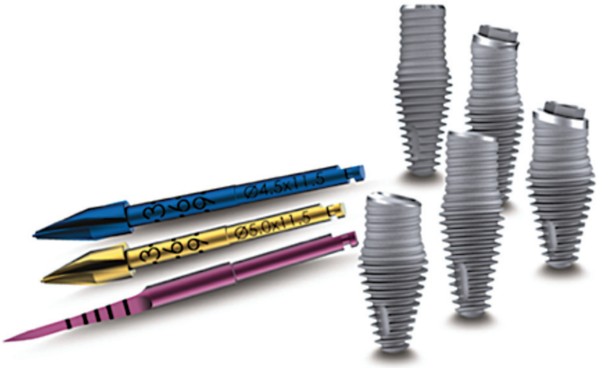

Supplementary Fig. S1 Various Inverta implants and drill sequence.

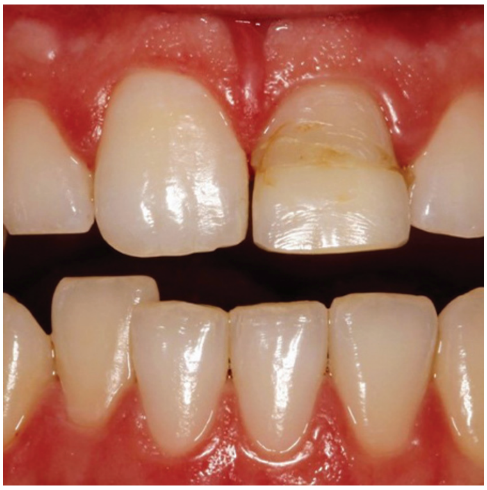

Supplementary Fig. S2 Preoperative clinical image used for pink esthetic score (PES) evaluation of the failing left maxillary central incisor.

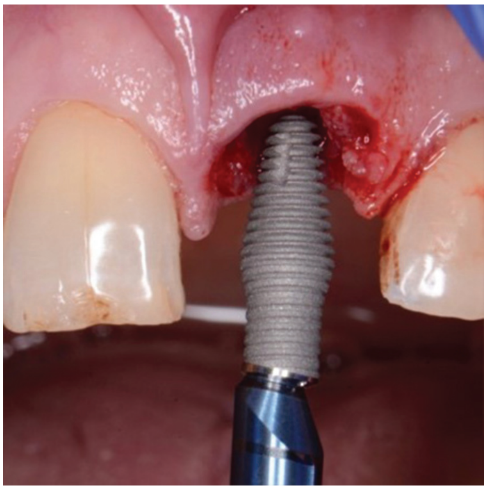

Supplementary Fig. S3 Labial view of an Inverta implant during placement.

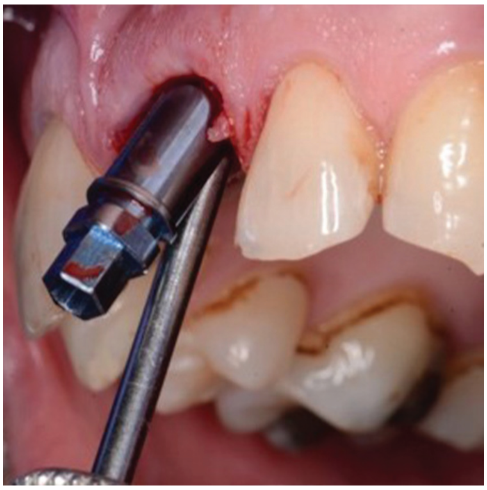

Supplementary Fig. S4 The Inverta implant placement showing angulation of the placement enabled by internal angle correction (Co-Axis).

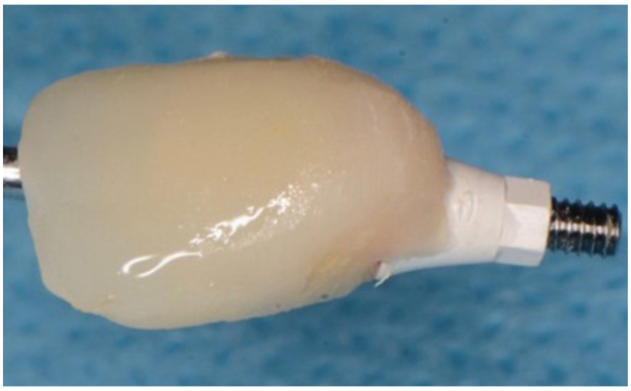

Supplementary Fig. S5 Provisional screw-retained restoration ready for placement.

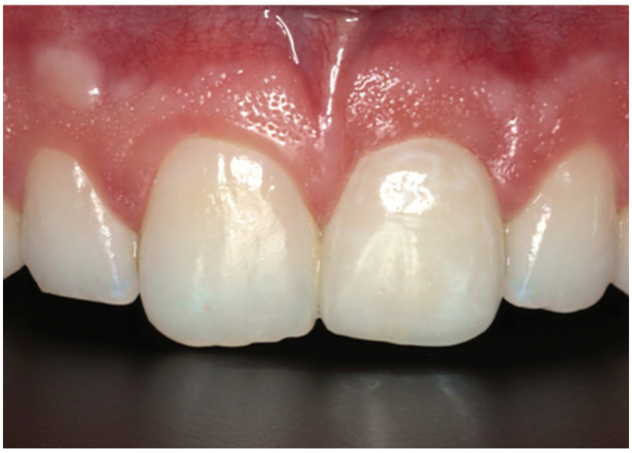

Supplementary Fig. S6 Postoperative clinical image used for pink esthetic score (PES) evaluation of the provisional restoration following placement of the provisional restoration demonstrating natural esthetics.

 © 2025. The Author(s).

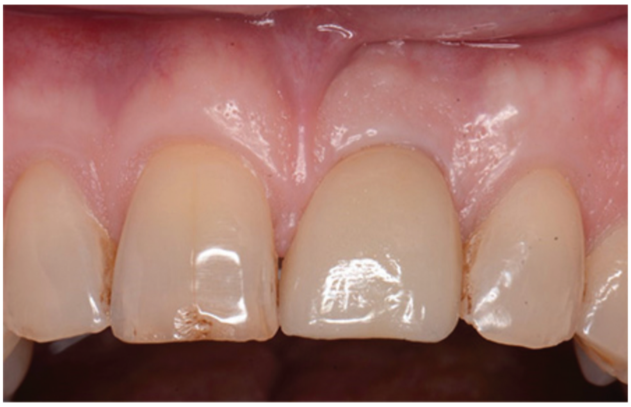

Supplementary Fig. S7 Clinical presentation following placement of the final restoration demonstrating natural emergence and soft tissue profile.

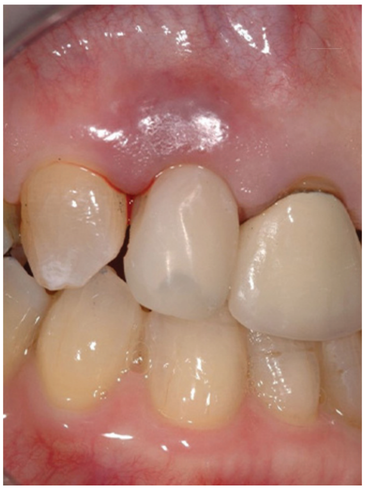

Supplementary Fig. S8 Preop clinical image of the failing lateral incisor with gingival inflammation on the facial aspect of the ridge.

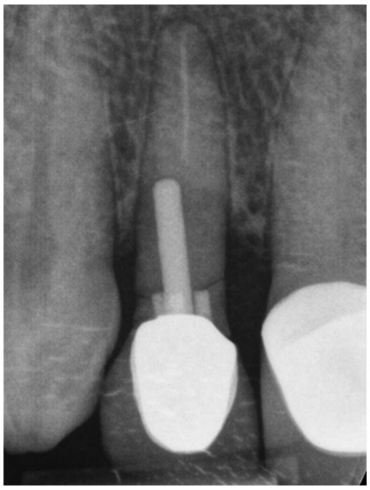

Supplementary Fig. S9 Preop radiograph of the lateral incisor that had prior endodontic treatment and was previously restored demonstrating a horizontal fracture apical to the crown margin.

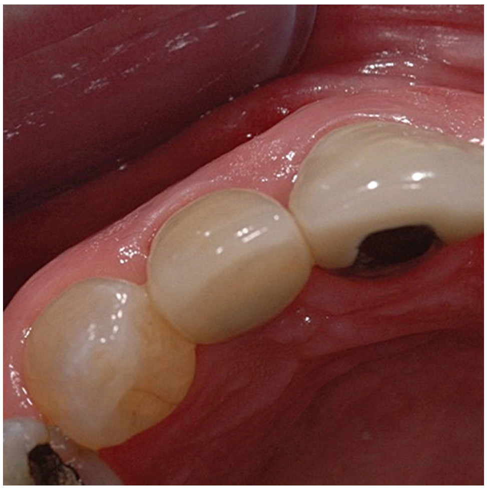

Supplementary Fig. S10 Postop ridge volume following placement of the final restoration demonstrating a natural contour to the ridge in relation to the adjacent natural teeth.

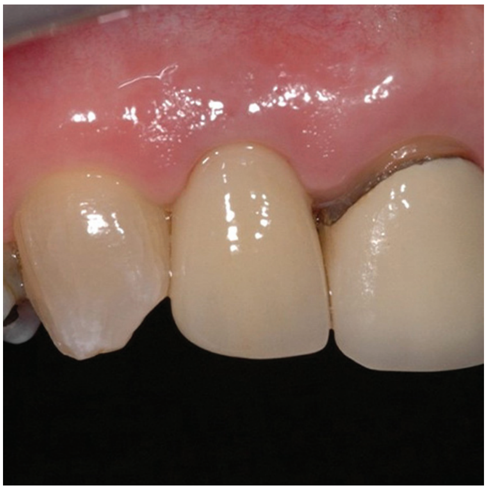

Supplementary Fig. S11 Postop labial view after placement of the final restoration demonstrating natural esthetics and contour of the ridge.

 © 2025. The Author(s).

Supplementary Table S1 Study key metrics

Column 1	Column 2	Column 3	Column 4
Total cases	31.0		
	Central	Lateral	Canine
Percentage tooth type	54.8	25.8	19.4
Mean age (combined)	58.8		
	Male	Female	
Mean age	53.8	69.2	
% sex	67.74	32.26	
Mean LBT preop (mm)	0.7		
Mean LBT postop (mm)	2.3		
Mean increase in LBT (mm)	1.7		
Range (mm)	0–2.1		
	Intact	Defective	Missing
Pre-op labial bone	83.9	12.9	3.2
ITV Ncm (mean)	58		
ITV Ncm (range)	10–100		
PES range	5–14		
PES preop mean	10		
PES postop mean	12		

Abbreviations: ITV, insertion torque value; LBT, lateral bone thickness; PES, pink esthetic score.

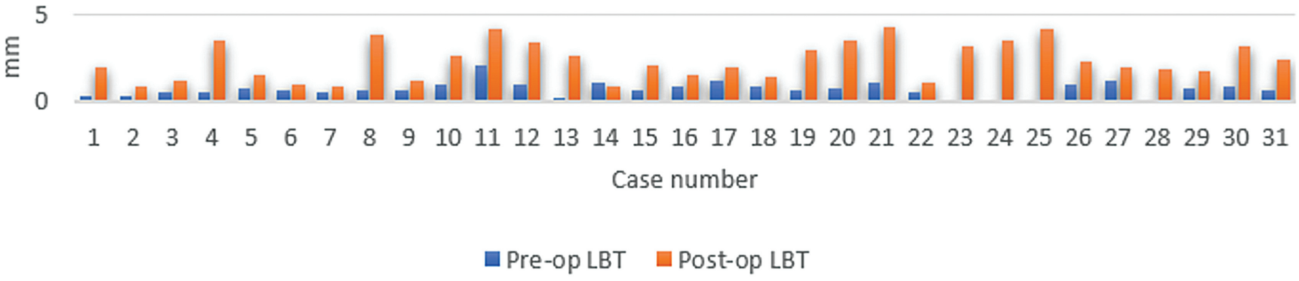

Supplementary Fig. S12 Preop vs. postop labial bone thickness (LBT).

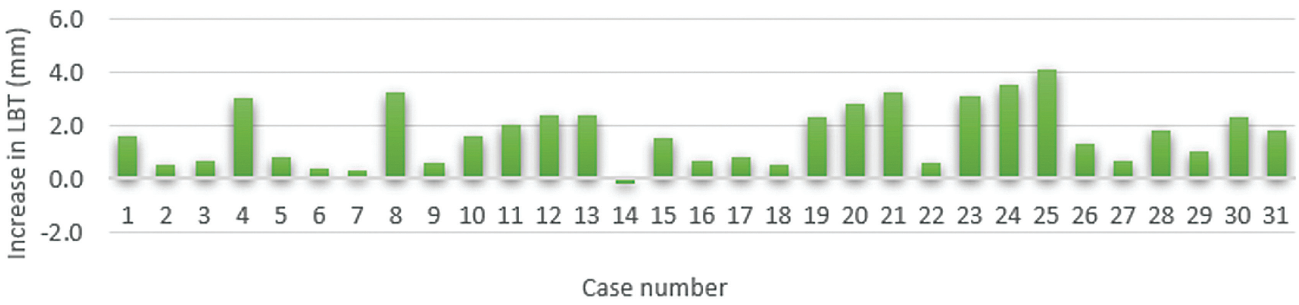

Supplementary Fig. S13 Increase observed in labial bone thickness (LBT).

 © 2025. The Author(s).

Supplementary Table S2 Statistical analysis of group means data

Labial plate thickness (LBT) preop (mm)	Labial plate thickness (LBT) postop (mm)
0.3	1.9
0.3	0.8
0.5	1.2
0.5	3.5
0.7	1.5
0.6	1.0
0.5	0.8
0.6	3.8
0.6	1.2
1	2.6
2.1	4.1
1	3.4
0.2	2.6
1.1	0.9
0.6	2.1
0.8	1.5
1.2	2
0.9	1.4
0.6	2.9
0.7	3.5
1.1	4.3
0.5	1.1
0	3.1
0	3.5
0	4.1
1	2.3
1.2	1.9
0.0	1.8
0.7	1.7
0.8	3.1
0.6	2.4

Supplementary Table S3 *F*-test two-sample for variances

	Variable 1	Variable 2
Mean	0.667741935	2.322580645
Variance	0.194258065	1.174473118
Observations	31	31
df	30	30
F	0.165400179	
p ($F \leq f$) one-tail	2.02449E-06	
F critical one-tail	0.543220913	

Abbreviation: df, degrees of freedom.
Result: Variances were unequal proceeding with *t*-test assuming unequal variances.

Supplementary Table S4 *t*-Test two-sample assuming unequal variances

	Variable 1	Variable 2
Mean	0.667741935	2.322580645
Variance	0.194258065	1.174473118
Observations	31	31
Hypothesized mean difference	1.65483871	
df	40	
t-Stat	–15.75097467	
p ($T \leq t$) one-tail	4.76993E-19	
t-Critical one-tail	1.683851013	
p ($T \leq t$) two-tail	9.53987E-19	
t-Critical two-tail	2.02107539	

Abbreviation: df, degrees of freedom.
Result: Perform two-tail test (Tstat $< -t$ or Tstat $> -15.75 << -2.02$, i.e., t Stat $<< -t$ critical.
According to the *t*-test, there is significant difference between the groups ($t = 15.75$, $a = 0.05$).

 © 2025. The Author(s).
